# The COVID-19 Pandemic and Goals-of-Care Conversations in Veterans Health Administration Clinics

**DOI:** 10.1001/jamanetworkopen.2025.15980

**Published:** 2025-06-16

**Authors:** Amy M. Linsky, Benjamin E. Canter, Mark Glickman, Shirley Qian, Samantha K. Ryan, Michael Still, Terri R. Fried, Renda Soylemez Wiener

**Affiliations:** 1 Center for Health Optimization and Implementation Research, Veterans Affairs Boston Healthcare System, Boston, Massachusetts; 2 Department of Medicine, Boston University Chobanian and Avedisian School of Medicine, Boston, Massachusetts; 3 New England Geriatric Research Education and Clinical Center, Veterans Affairs Boston Healthcare System, Boston, Massachusetts; 4 Department of Occupational Therapy, Sargent College of Health and Rehabilitation Sciences, Boston University, Boston, Massachusetts; 5 Department of Statistics, Harvard University, Cambridge, Massachusetts; 6 Pain, Research, Informatics, Multi-Morbidities, and Education Center, Veterans Affairs Connecticut Healthcare System, West Haven; 7 Department of Medicine, Yale School of Medicine, New Haven, Connecticut

## Abstract

**Importance:**

The onset of the COVID-19 pandemic created urgency for advance care planning, including documenting goals-of-care conversations (GoCCs), while seismically disrupting usual health care delivery. Characterizing trends in GoCC rates during the pandemic can provide insight into the extent to which health care systems prioritized advance care planning in the face of competing clinical demands, shifts to telemedicine, and staffing shortages.

**Objective:**

To determine how the COVID-19 pandemic was associated with changes in outpatient first-ever GoCCs.

**Design, Setting, and Participants:**

Retrospective cohort study of patients eligible for their first-ever documented GoCC in outpatient clinics at 123 US Veterans Health Administration facilities nationwide from March 2019 to February 2023.

**Exposure:**

COVID-19 pandemic.

**Main Outcomes and Measures:**

National- and facility-level weekly GoCC rates, defined as number of first-ever documented GoCCs per 100 000 outpatient appointments. Secondary analyses examined associations between facility-level characteristics and facility GoCC rates.

**Results:**

Of 5 027 956 patients nationally, 124 216 (2.5%) had a first-ever outpatient GoCC during the study period (facility-level range: 0.01%-26.3%). The mean (SD) weekly national first-ever outpatient GoCC rate was 99.6 (12.1) in the year preceding the pandemic. At pandemic onset, mean weekly outpatient GoCC rates dropped to a nadir of 74.1 (week of March 21, 2020), then sharply increased, peaking at 177.4 (week of April 18, 2020), before steadily declining to pre–COVID-19 rates and ending with a COVID year 3 mean (SD) of 96.6 (11.5). At the facility level, 29 of 123 facilities (23.6%) significantly increased outpatient GoCC rates in the early pandemic and maintained or further improved through COVID year 3, with significant rate increases pre-COVID to COVID year 3.

**Conclusions and Relevance:**

In this retrospective cohort study of outpatient GoCC rates, the early COVID-19 pandemic was associated with initial disruption and then with increased first-ever outpatient GoCC rates nationally. Despite unprecedented challenges to health care delivery, several facilities increased GoCC rates during the first COVID-19 surge and maintained increased rates through 2023. These facilities could serve as models for best practices to improve advance care planning.

## Key Points

**Question:**

Was the COVID-19 pandemic associated with initiation of more goals-of-care conversations (GoCCs) in the Veterans Health Administration?

**Findings:**

This cohort study of more than 5 million patients from 2019 to 2023 found national weekly GoCC rates (number of GoCCs per 100 000 appointments) dropped from a pre-COVID mean of 99.6 to a nadir of 74.1 (COVID week 4), then spiked to 177.4 (week 8), before gradually returning to prepandemic levels. One-quarter of facilities (29 of 123) significantly increased GoCC rates early in the pandemic and maintained increases through 2023.

**Meaning:**

These findings suggest that large-scale crises can catalyze health care delivery innovations; facilities that sustainably increased GoCCs may serve as case studies to identify and disseminate best practices.

## Teaser

This cohort study investigates whether the COVID-19 pandemic was associated with changes in outpatient first-ever goals-of-care conversations among patients treated at the Veterans Health Administration (VA).

## Introduction

Advance care planning—defined by an expert consensus panel as “a process that supports adults at any age or stage of health in understanding and sharing their personal values, life goals and preferences regarding future medical care”^1^—is critical to achieving patient-centered care.^2,3,4^ A central component of advance care planning is having and documenting goals-of-care conversations (GoCCs).^1,2,3^ During GoCCs, health care practitioners elicit patients’ values regarding what is most important, and the patient and practitioner collaborate to make patient-centered, goal-concordant decisions regarding use of life-sustaining treatment (LST; eg, cardiopulmonary resuscitation and mechanical ventilation).^3^ GoCCs ideally are proactive, occurring before there is an urgent need or acute illness during which patients may not be able to communicate their preferences.^3^ Proactive GoCCs are recommended by prominent organizations such as the National Academy of Medicine, American College of Physicians, and National Quality Forum,^4,5,6^ and have been shown to optimize health outcomes,^7^ reduce family distress,^8^ and promote trust in clinicians.^9^ Yet despite these recommendations and clear benefits, advance care planning is underused.^10^ For example, in the national Veterans Health Administration (VA), fewer than 2% of all veterans and only 16% of veterans with high predicted mortality had a documented GoCC as of January 2020.^11^

The onset of the COVID-19 pandemic amplified the importance of proactive GoCCs, especially among patients without prior documented advance care planning. Early in the pandemic, COVID-19 infections commonly resulted in respiratory failure and death.^12^ Professional guidelines and lay press encouraged proactive, documented GoCCs to ensure goal-aligned care and ethical allocation of limited resources (eg, ventilators).^3^ Meanwhile, isolation protocols required practitioners to pivot to telehealth,^13^ a modality that some practitioners and patients felt uncomfortable using for sensitive conversations like GoCCs at the start of the pandemic.^14,15^ Finally, the pandemic exacerbated known barriers to GoCCs, including staffing,^16^ time constraints,^16^ and discomfort with prognostication.^17^

It is unknown how these rapid changes to health care delivery impacted GoCC frequency. Understanding how COVID-19–related changes affected proactive outpatient GoCC rates could help health systems prepare for future pandemics or other events that disrupt health care delivery. Moreover, facility-level analyses could identify sites with robust and lasting increases in GoCCs in response to the pandemic; these sites could serve as exemplars of best practices that could be disseminated to other sites to improve advance care planning. In this study, we assessed VA national- and facility-level trends from 2019 to 2023 to ascertain if the pandemic was associated with rates of documented outpatient first-ever GoCCs. We hypothesized that GoCC rates would increase in anticipation of COVID-19 surges and that some facilities would sustain increased GoCC rates for years after pandemic onset.

## Methods

The VA central institutional review board (IRB) conducted a limited review and determined that this retrospective cohort study, which analyzed previously collected electronic health record data, was exempt from IRB oversight and the need for informed consent. Study activities were overseen by the Research and Development Committees at VA Boston, VA Bedford, and VA Connecticut Healthcare Systems. This study followed the Strengthening the Reporting of Observational Studies in Epidemiology (STROBE) reporting guideline for cohort studies.

### Study Population and Setting

We used the VA Corporate Data Warehouse to identify patients with any completed outpatient medical, surgical, or mental health visit (in-person or telehealth) from March 2019 to February 2023. We focused on GoCCs conducted in outpatient settings because they are more likely to represent proactive advance care planning than GoCCs held in emergency department or inpatient settings. We included medical, surgical, and mental health clinics because they represent major domains of outpatient care provided in the VA and opportunities to conduct and document GoCCs. Outpatient visits from all VA medical centers and their associated community-based outpatient clinics were included, except those located in US territories (Manila, Philippines; San Juan, Puerto Rico; and Agana Heights, Guam) or that did not have data available for the full study period due to early transition to a new electronic health record (Spokane, Washington; Walla Walla, Washington; Columbus, Ohio; Roseburg, Oregon; and White City, Oregon). Visits that occurred in community-based outpatient clinics were aggregated to the parent medical center facility.

Since GoCCs are uncommon and often less warranted in younger people, who tend to be in better health, patients aged less than 50 years were excluded.^11,18^ We excluded patients with prior GoCC(s) documented with VA’s standardized LST note template (described in the subsequent section) and those who used VA long-term care or home-based primary care due to prior initiatives to increase GoCCs in those settings.^19,20^

### Primary Outcome

Our primary outcomes were national- and facility-level weekly GoCC rates, defined as number of first-ever documented GoCCs per 100 000 outpatient appointments. In our statistical modeling, we included these outcomes as binomial counts (ie, total number of GoCCs in a week out of the total number of outpatient appointments). For the purposes of this study, documented GoCCs were those captured in the VA’s required LST note template. The LST note template includes 4 required fields to document GoCC content: (1) veteran’s decision-making capacity, (2) veteran’s values and goals of care, (3) life-sustaining treatment plan (eg, code status and preference regarding mechanical ventilation), and (4) informed consent for LST plan. Other fields are optional, including documentation of surrogate decision-maker, veterans’ understanding of their health status, review of other advance care planning documents, and GoCC participants. Prior work^21^ has confirmed the content validity of LST notes for capturing GoCCs through detailed electronic health record review.

### Primary Exposure

Our primary exposure was the COVID-19 pandemic. We calculated weekly GoCC rates during the COVID-19 pandemic and examined GoCC rates across different phases of the COVID pandemic.

We divided the study period into distinct analytic phases, separately defined for national- and facility-level analyses (Table 1). All phase start and end dates were based on 52 weeks per year because analyses were of weekly rates. The national-level analyses included 4 phases: the year preceding the COVID-19 pandemic (pre-COVID; March 2, 2019, to February 28, 2020), first year of the pandemic (COVID Y1; February 29, 2020, to February 26, 2021), second year of the pandemic (COVID Y2; February 27, 2021, to February 25, 2022), and third year of the pandemic (COVID Y3; February 26, 2022, to February 24, 2023).

**Table 1.** Definitions of Study Period Phases Used in Analyses

Phases for national analyses/phases for facility-level analyses	Phase name	Phase dates^a^
Year preceding the COVID-19 pandemic	Pre-COVID	March 2, 2019, to February 28, 2020
First y of COVID-19 pandemic		
Anticipatory period before first local COVID-19 surge^b^	COVID Y1a	February 29, 2020, to local risk elevation date
First local COVID-19 surge^b^	COVID Y1b	Local risk elevation date to 4 wk post–local risk elevation date
Rest of pandemic y 1 following the first local COVID-19 surge^b^	COVID Y1c	4 wk post–local risk elevation date to February 26, 2021
Second y of COVID-19 pandemic	COVID Y2	February 27, 2021, to February 25, 2022
Third y of COVID-19 pandemic	COVID Y3	February 26, 2022, to February 24, 2023

^a^ Phase start and end dates are based on 52 weeks per year, because analyses were of weekly rates.

^b^ We used data from the US Centers for Disease Control and Prevention COVID-19 community levels to identify each Veterans Health Administration facility’s county’s first COVID-19 surge, based on first local risk elevation date (ie, first transition from low to medium risk based upon county-level COVID-19 cases, admissions, and hospital bed capacity).

The facility-level analyses further divided the first pandemic year (COVID Y1) into 3 subphases based on local COVID-19 surge timing, which we determined using COVID-19 community levels reported by the Centers for Disease Control and Prevention (CDC).^22^ Specifically, we used CDC COVID-19 community levels data to identify the first COVID-19 surge occurring in each VA facility’s county, based on first local risk elevation date (ie, first transition from low to medium risk). The CDC developed COVID-19 community levels using a combination of 3 metrics: new COVID-19 admissions per 100 000 population in the past 7 days, the percentage of staffed inpatient beds occupied by COVID-19 patients, and total new COVID-19 cases per 100 000 population in the past 7 days. The COVID-19 community level was determined by the higher of the new admissions and inpatient beds metrics, based on the level of new cases per 100 000 population in the past 7 days. New COVID-19 admissions and the percentage of staffed inpatient beds occupied represented the current potential for strain on local health systems. Data on new cases acted as an early warning indicator of potential increases in health system strain in the event of a COVID-19 surge. Using these data, the COVID-19 community level was classified as low, medium, or high.

For the purposes of our facility-level analyses, COVID Y1 was divided into the following subphases: the anticipatory period before the first local COVID-19 surge (COVID Y1a; from February 29, 2020, until the date of local risk elevation shift from low to medium risk), the first local COVID-19 surge (COVID Y1b; from local risk elevation date to 4 weeks thereafter), and the rest of pandemic year 1 following the first local COVID-19 surge (COVID Y1c; four weeks after local risk elevation date through February 26, 2021).

### Other Variables

We examined facility-level variables that we hypothesized a priori might change rates of first-ever outpatient GoCCs. These included facility complexity (a 5-level VA rating scale that takes into account patient volume and risk, level of teaching and research, number of specialists, and presence of intensive care units within the facility); geographic region (defined by US census regions); rurality (designated by the VA’s Office of Rural Health based upon the percentage of veterans assigned to a given facility who reside in areas considered rural or highly rural, as defined by the US Census Bureau’s Rural-Urban Commuting Area designations); geriatrics clinic presence (defined as at least 50 outpatient encounters in a geriatrics clinic in 2020); palliative care clinic presence (defined as at least 50 outpatient encounters in a palliative care clinic in 2020); and logarithm of local COVID-19 case counts.

### Statistical Analysis

We used additive logistic regression to estimate the national weekly probability of a patient having a GoCC based on the binomial count of first GoCCs across all facilities and a smoothing spline over time (by week).^23,24^ We used a χ^2^ test or Fisher exact test when appropriate, based on the raw counts to compare the GoCC rates between pre-COVID and COVID Y3.

We fit a facility-specific additive logistic regression model, with the facility-level binomial outcome and a separate smoothing spline of week for each facility, controlling for all other variables. We simulated 10 000 facility-specific splines from a multivariate normal distribution centered on the estimated facility-specific curves, using the model’s covariance matrix. Curves were converted to probabilities via an inverse-logit transformation. To determine significant changes in adjusted GoCC rates between phases, we computed mean estimated GoCC probability differences between each phase for each simulated curve. Specifically, we compared GoCC rates between the prepandemic year (pre-COVID) and the anticipatory period before the first local surge (COVID Y1a), between pre-COVID and the peak of the first local surge (COVID Y1b), between pre-COVID and COVID Y3, and between COVID Y1b and COVID Y3. The *P* value for phase comparisons was twice the tail probability, based on the 10 000 differences being compared with 0. All tests were 2-tailed and were performed at the family-wise significance level of 5% after applying the Holm adjustment procedure to account for multiple tests in both national and facility-specific analyses. We applied the Holm adjustment procedure^25^ for multiple tests in both national and facility-specific analyses.

## Results

### National

From March 2019 through February 2023, across the 123 included VA medical centers nationally, 124 216 of 5 027 956 patients (2.5%) had a first-ever documented outpatient GoCC. During the study period, there were 151 040 000 completed outpatient appointments in eligible clinics, including 92 471 714 (61.2%) medical, 26 701 544 (17.7%) surgical, and 31 861 760 (21.1%) mental health visits.

The Figure shows national weekly outpatient first-ever GoCC rates per 100 000 appointments. The mean (SD) weekly rate was 99.6 (12.1) during pre-COVID, 114.8 (23.2) in COVID Y1, 94.2 (15.7) in COVID Y2, and 96.6 (11.5) in COVID Y3. Rates varied seasonally throughout the study period, with summer peaks and winter nadirs.

**Figure.**
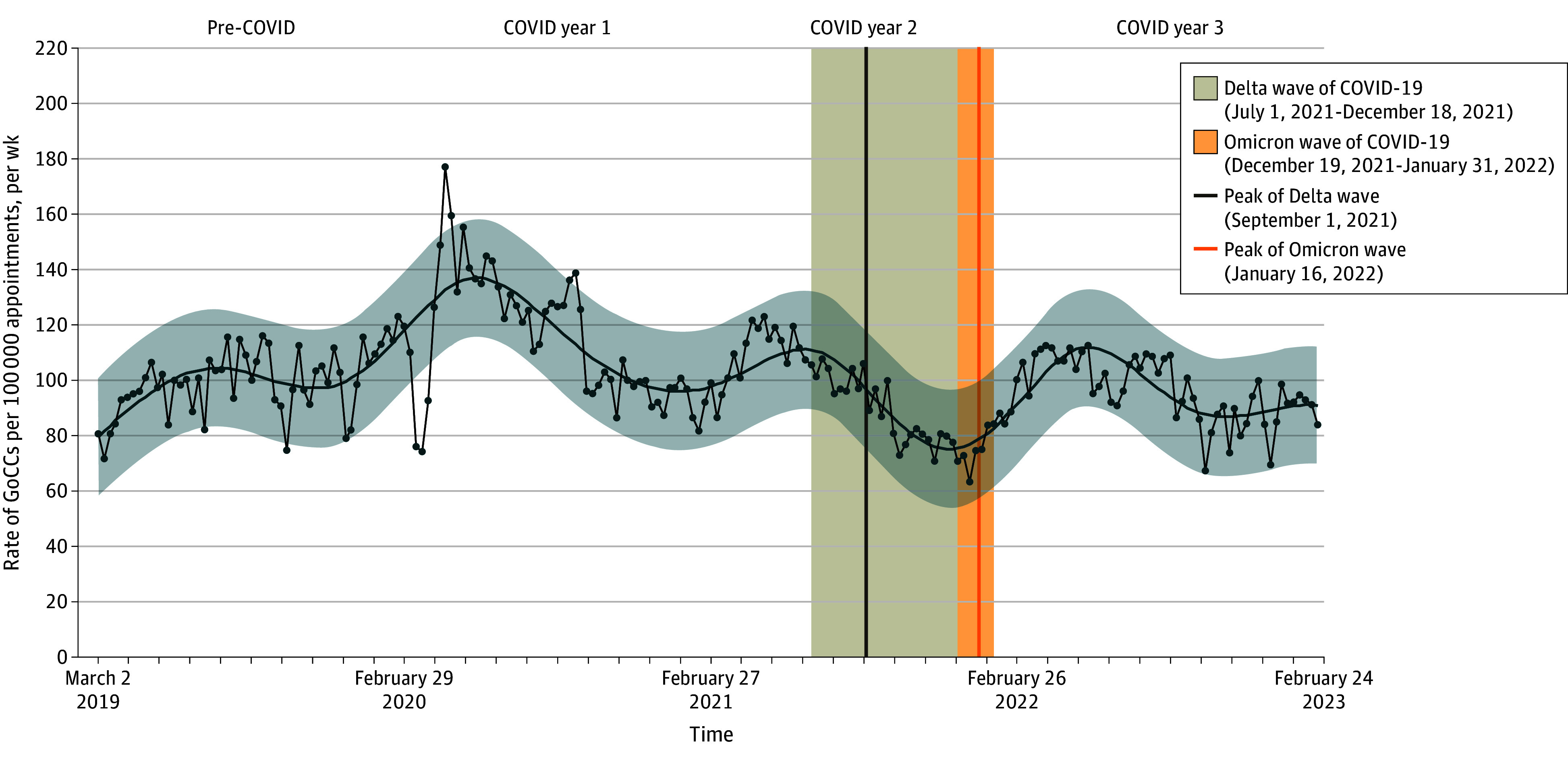
National Trends in First-Ever Documented Goals-of-Care Conversation (GoCC) Rates in Veterans Health Administration Outpatient Settings, March 2019 to February 2023

Weekly GoCC rates gradually increased from 80.6 to 123.2 during pre-COVID. In the early pandemic, mean GoCC rates fluctuated beyond the 95% prediction interval during weeks 3 to 5 and 8 to 9 of COVID Y1, dropping to a minimum of 74.1 (week of March 21, 2020), and then more than doubling to 177.4 (week of April 18, 2020). Rates decreased thereafter during 2020. The sharp fluctuation in GoCC rates observed during the first COVID wave was not observed with subsequent surges (eg, Delta or Omicron) (Figure).

### Facility Level

Characteristics of the 123 VA facilities included in our analyses are shown in Table 2. Across facilities, the proportion of patients who had a documented first-ever outpatient GoCC during the study period ranged from 0.1% to 26.3%, with only 11 facilities exceeding 5%.

**Table 2.** Characteristics of the Veterans Health Administration Facilities (N = 123)

Characteristics	Facilities, No. (%)
Facility complexity	
1a, High complexity	38 (30.89)
1b, High complexity	21 (17.07)
1c, High complexity	20 (16.26)
2, Medium complexity	18 (14.63)
3, Low complexity	26 (21.14)
Region	
Midwest	24 (19.51)
Northeast	22 (17.89)
South	49 (39.84)
West	28 (22.76)
Presence of palliative care clinic	113 (91.87)
Presence of geriatric care clinic	109 (88.62)
Patients classified as rural, mean (SD), %	37.1 (23.8)
Logarithm of weekly COVID-19 cases, mean (SD)^a^	
Pre-COVID	0.04 (0.17)
Anticipatory (Y1a)	4.07 (0.97)
Local surge (Y1b)	5.89 (1.34)
Remainder of COVID Y1 (Y1c)	6.81 (1.28)
COVID Y2	7.09 (1.39)
COVID Y3	6.33 (1.47)

^a^ Pre-COVID: March 2, 2019, to February 28, 2020; anticipatory: February 29, 2020, to local risk elevation date; local surge: local risk elevation date to 4 weeks post–local risk elevation date; remainder of COVID Y1: 4 weeks post–local risk elevation date to February 26, 2021; COVID Y2: February 27, 2021, to February 25, 2022; COVID Y3: February 26, 2022, to February 24, 2023.

GoCC rates varied across facilities during pandemic phases. Twenty-nine of 123 VA facilities (23.6%) significantly increased GoCC rates from pre-COVID to the COVID Y1 anticipatory period and/or first local surge (COVID Y1a and/or COVID Y1b) and sustained (16 facilities) or further increased (13 facilities) GoCC rates through COVID Y3, such that GoCC rates were significantly higher in COVID Y3 than pre-COVID (Table 3). Overall, 60 facilities (48.7%) from all geographic regions significantly increased GoCC rates from pre-COVID to COVID Y3, with a mean (SD) increase of 83.5 (113.8) GoCCs per 100 000 outpatient appointments over the study period (eTable 1 in Supplement 1). By contrast, a minority of facilities (17 of 123 [13.8%]) significantly decreased GoCC rates, with a mean (SD) decrease of 356.6 (765.1) GoCCs per 100 000 outpatient appointments over the study period. Among the explored facility characteristics (eTable 2 in Supplement 1), only geriatrics clinic presence was significantly associated with increased GoCC rates from pre-COVID to COVID Y3 (χ^2^_1_ = 14.891; *P* = .002).

**Table 3.** Facility-Level Patterns in Rates of Goals-of-Care Conversations (GoCC) Between Pandemic Phases

Trend group	Change in GoCC rates from		Facilities, No.	No. of facilities with each pattern of change in GoCC rates from pre-COVID to COVID-Y3^c^		
	Pre-COVID to anticipatory period (COVID-Y1a) and/or 1st local surge (COVID Y1b)^a^^,^^b^^,^^c^	1st Local surge (COVID-Y1b) to COVID-Y3^a^^,^^b^^,^^c^				
				Decrease	No detectable change	Increase
1	Decrease	Decrease	3	3	0	0
2	Decrease	No detectable change	9	7	2	0
3	Decrease	Increase	3	0	0	3
4	No detectable change	Decrease	3	2	1	0
5	No detectable change	No detectable change	33	0	26	7
6	No detectable change	Increase	15	0	2	13
7	Increase	Decrease	23	5	10	8
8	Increase	No detectable change	21	0	5	16^d^
9	Increase	Increase	13	0	0	13^d^
Total	NA	NA	123	17 (13.8%)	46 (37.4%)	60 (48.7%)

Abbreviation: NA, not applicable.

^a^ All facility increases and decreases are significant at a *P* < .05 level. No detectable change indicates no significant change between phases.

^b^ There were no instances where anticipatory and local surge phases significantly changed in opposite directions compared with pre-COVID-19 (eg, pre-COVID-19 to COVID Y1a significantly increased and pre-COVID-19 to COVID Y1b significantly decreased, or vice versa). If there is only a significant increase between pre-COVID and 1 of these phases, no detectable change existed between pre-COVID and the second phase.

^c^ Pre-COVID: March 2, 2019, to February 28, 2020; anticipatory: February 29, 2020, to local risk elevation date; local surge: local risk elevation date to 4 weeks post–local risk elevation date; COVID-Y3: February 26, 2022, to February 24, 2023.

^d^ Indicates GoCC rates were significantly higher in COVID Y3 than pre-COVID.

## Discussion

The onset of the COVID-19 pandemic was associated with an initial decrease and then with an increase in GoCC rates in the national VA. The spike in GoCC rates observed during the first COVID-19 surge was not sustained over time nationally and did not occur in subsequent waves. Notably, a quarter of facilities significantly increased GoCC rates leading up to or during the first local COVID-19 surge and maintained or further increased rates through 2023, resulting in higher GoCCs in COVID Y3 than pre-COVID. Even if unable to increase rates during the challenges of the early pandemic, by 2023 nearly half of facilities in the national VA system had achieved higher GoCC rates compared with pre-COVID, including in geographic regions where GoCC rates are historically low.^26^

The sharp decrease in GoCC rates in March 2020 may reflect competing clinical demands in the early pandemic and a shift to telehealth; both clinicians and veterans initially experienced discomfort with GoCCs held virtually.^14^ Meanwhile, the sharp rise in outpatient GoCC rates nationally around the first surge may reflect quality improvement efforts prompted by anticipated or actual local rises in COVID-19 cases, responding to calls urging advance care planning.^2,3^

Sustaining early gains when initial quality improvement efforts wane is a challenge.^11^ As the pandemic evolved, COVID-19 treatment and outcomes improved, potentially dampening the perceived urgency of proactive GoCCs. Yet, the fact that a quarter of facilities increased GoCC rates in the early pandemic and sustained or further increased these rates through 2023 suggests that strategies to catalyze GoCCs can remain impactful beyond initial implementation. These facilities could potentially serve as case studies of best practices that could be spread to lower-performing facilities. For example, we found that presence of geriatrics clinics was associated with a sustained increase in GoCC rates from pre-COVID to COVID Y3. However, it is unknown what special efforts geriatrics clinics may have made to encourage GoCCs over the course of the pandemic; future qualitative research could explore this question.

While the sustained, statistically significant increases in GoCC rates achieved in some facilities is promising, a notable finding of our study is that proactive GoCCs were grossly underutilized in all facilities. Nationally, only 2.5% of veterans in our sample had a first-ever documented outpatient GoCC at any point in the study period, with only 11 of 123 facilities exceeding 5%. However, we acknowledge that it is impossible to ascertain what the right proportion of veterans with first-ever outpatient GoCCs should have been during the pandemic; undoubtedly our denominator included veterans whose excellent health status, age, or preferences regarding advance care planning meant that there was no immediate indication for a GoCC. In fact, the influx of several hundred thousand veterans into VA care during the study period as a result of the Promise to Address Comprehensive Toxics (PACT) Act of 2022, most of whom were under age 65,^27^ may have expanded the denominator of veterans eligible for first-ever GoCCs but not added much to the numerator, thus potentially blunting the increase in GoCCs we observed over the study period. It is also debatable which clinical settings represent the right opportunities for GoCCs. While outpatient advance care planning tends to occur in medical settings, prior initiatives have sought to engage other practitioner types and clinical disciplines in holding GoCCs, and the pandemic sparked new efforts in this arena.^2,28^ Our study confirmed that there is ample opportunity for a multidisciplinary approach to GoCCs: in our cohort, 21.1% of appointments were in mental health and 17.7% in surgical clinics, and indeed, there have been calls for both mental health practitioners and surgeons to engage in GoCCs.^29,30,31,32,33,34^ Exploring the extent to which advance care planning occurs in various outpatient clinical settings may be a fruitful area for future research.

### Limitations

This study has limitations. Results may not generalize to non-VA systems. However, using data from the largest nationally integrated health care system allowed us to comment on national GoCC trends associated with COVID-19-related challenges and opportunities. We captured only GoCCs documented using standardized LST note templates. It is highly likely that GoCCs occurred during the study period that were documented in other formats (eg, free text in a clinic progress note) or not documented at all, and as such, our estimates are likely lower than true GoCC rates. However, a key indicator of GoCC quality is easy retrievability of documentation,^4^ which was central to the VA’s decision to require documentation of GoCCs in LST notes; thus it is critical to understand rates of first-ever outpatient GoCCs documented in LST notes, as reported in this manuscript. We did not account for individual patient patterns over time, such as the possibility that a patient who did not have a GoCC in one week might be more (or less) likely to have one in a later week; including patient-specific trends would have added significant complexity and computing demands beyond the scope of this study. Finally, our analyses focused on how the COVID-19 pandemic changed rates of documented first-ever outpatient GoCCs, but did not shed light on how the pandemic affected the quality of these GoCCs, including the topics covered or the extent to which conversations accurately captured the values and preferences of patients for end-of-life care.

## Conclusions

Advance care planning is underutilized both within and outside the VA; increasing proactive outpatient GoCC rates remains critical despite the receding threat from COVID-19. While peak national outpatient first-ever GoCC rates achieved during the early pandemic were not sustained over time, several facilities successfully maintained GoCC increases achieved in the early pandemic, suggesting that times of unprecedented challenges to the health care system can also catalyze sustainable innovations in advance care planning. Notably, the pandemic may have stimulated not only first-ever outpatient GoCCs, but also GoCCs at other points on a patient’s trajectory, such as first-ever GoCCs occurring in the context of a hospitalization, or revisiting GoCCs that had been previously documented. Future research could further characterize the impact of the pandemic on the full spectrum of GoCCs.

## Notes

Supplement 1. **eTable 1. **Facility-Level Changes in Rates of Goals-of-Care Conversations Per 100 000 Appointments Between Pandemic Phases **eTable 2. **Differences in Facility-Level Characteristics Between Facilities With Significant Increases vs Significant Decreases in Goals of Care Conversation Rates Throughout the COVID-19 Pandemic

Supplement 2.
Data Sharing Statement
