# Supplementary material for: The COVID-19 Pandemic and Goals-of-Care Conversations in Veterans Health Administration Clinics
Source: JAMA Netw Open. 2025 Jun 16;8(6):e2515980. doi: 10.1001/jamanetworkopen.2025.15980 (PMC12171934; doi:10.1001/jamanetworkopen.2025.15980)
Supplement: Supplement 1. — eTable 1. Facility-Level Changes in Rates of Goals-of-Care Conversations Per 100 000 Appointments Between Pandemic Phases eTable 2. Differences in Facility-Level Characteristics Between Facilities With Significant Increases vs Significant Decreases in Goals of Care Conversation Rates Throughout the COVID-19 Pandemic [file jamanetwopen-e2515980-s001.pdf]

Supplemental Online Content

Linsky AM, Canter BE, Glickman M, et al. The COVID-19 pandemic and goals-of-care conversations in Veterans Health Administration clinics. *JAMA Netw Open*. 2025;8(6):e2515980. doi:10.1001/jamanetworkopen.2025.15980

eTable 1. Facility-Level Changes in Rates of Goals-of-Care Conversations Per 100 000 Appointments Between Pandemic Phases
eTable 2. Differences in Facility-Level Characteristics Between Facilities With Significant Increases vs Significant Decreases in Goals of Care Conversation Rates Throughout the COVID-19 Pandemic

This supplemental material has been provided by the authors to give readers additional information about their work.

© 2025 Linsky AM et al. *JAMA Network Open*.

eTable 1. Facility-Level Changes in Rates of Goals-of-Care Conversations Per 100 000 Appointments Between Pandemic Phases

Trend Group	Change in GoCC rates from Pre-COVID to Anticipatory Period (COVID-Y1a) and/or First Local Surge (COVID-Y1b)*†‡	Change in GoCC rates from First Local Surge (COVID-Y1b) to COVID-Y3*†‡	Facilities (n)	Change in GoCC rates from Pre-COVID to COVID-Y3‡						
				Decrease			No Detectable Change	Increase		
				Facilities (n)	Mean Change in GoCC Rate	SD		Facilities (n)	Mean Change in GoCC Rate	SD
1	Decrease	Decrease	3	3	-1635.3	1297.0	0	0		
2	Decrease	No Detectable Change	9	7	-95.3	81.3	2	0		
3	Decrease	Increase	3	0			0	3	113.3	104.5
4	No Detectable Change	Decrease	3	2	-90.2	21.3	1	0		
5	No Detectable Change	No Detectable Change	33	0			26	7	36.4	17.1
6	No Detectable Change	Increase	15	0			2	13	141.7	162.4
7	Increase	Decrease	23	5	-61.9	29.8	10	8	117.7	195.6
8	Increase	No Detectable Change	21	0			5	16	36.3	17.8
9	Increase	Increase	13	0			0	13	81.0	61.0
**Total**			**123**	**17 (13.8%)**	**-356.6**	**765.1**	**46 (37.4%)**	**60 (48.7%)**	**83.5**	**113.8**

* All facility increases and decreases, are significant at a $p<0.05$ level. “No detectable change” indicates no significant change between phases.

† There were no instances where Anticipatory Period (COVID-Y1a) and First Local Surge (COVID-Y1b) phases significantly changed in opposite directions compared to Pre-COVID (e.g., Pre-COVID to COVID-Y1a significantly increased and Pre-COVID to COVID-Yb significantly decreased, or vice versa). If there was only a significant increase between Pre-COVID and one of these phases, no detectable change existed between Pre-COVID and the second phase.

‡ Pre-COVID: 3/2/2019–2/28/2020; Anticipatory Period (COVID-Y1a): 2/29/2020–Local risk elevation date; First Local Surge (COVID-Y1b): Local risk elevation date–4 weeks post-local risk elevation date; COVID-Y3: 2/26/2022–2/24/2023.

© 2025 Linsky AM et al. *JAMA Network Open*.

eTable 2. Differences in Facility-Level Characteristics Between Facilities With Significant Increases vs Significant Decreases in Goals of Care Conversation Rates Throughout the COVID-19 Pandemic

Variables	Anticipatory Period Before First Local Surge (COVID-Y1a) vs. Pre-COVID				First Local COVID-19 Surge (COVID-Y1b) vs. Pre-COVID				COVID-Y3 vs. Pre-COVID				COVID-Y3 vs. First Local COVID-19 Surge (COVID Y1b)			
	**Facilities with Increases in GoCC Rates (N=54)**	**Facilities with Decreases in GoCC Rates (N=13)**	**P-Value**	**Holm Adjusted**	**Facilities with Increases in GoCC Rates (N=56)**	**Facilities with Decreases in GoCC Rates (N=13)**	**P-Value**	**Holm Adjusted**	**Facilities with Increases in GoCC Rates (N=60)**	**Facilities with Decreases in GoCC Rates (N=17)**	**P-Value**	**Holm Adjusted**	**Facilities with Increases in GoCC Rates (N=31)**	**Facilities with Decreases in GoCC Rates (N=29)**	**P-Value**	**Holm Adjusted**
**Facility Complexity**			0.91	N/S			0.77	N/S			0.84	N/S			1.00	N/S
1a-high	43%	31%			41%	31%			33%	41%			39%	38%		
1b-high	20%	31%			20%	31%			18%	18%			13%	10%		
1c-high	8%	8%			13%	15%			17%	24%			10%	14%		
2-medium	12%	15%			11%	15%			17%	12%			23%	21%		
3-low	16%	15%			16%	8%			15%	6%			16%	17%		
**Region**			0.40	N/S			0.57	N/S			0.84	N/S			1.0000	N/S
Midwest	22%	8%			23%	8%			22%	12%			16%	21%		
Northeast	12%	8%			14%	8%			10%	12%			19%	17%		
South	41%	69%			41%	62%			45%	53%			42%	41%		
West	24%	15%			21%	23%			23%	24%			23%	21%		
**Percent of Patients Classified as Rural**	37%	39%	0.84	N/S	34%	42%	0.30	N/S	35%	39%	0.46	N/S	35%	41%	0.3471	N/S
**Presence of Palliative Care Clinic**	98%	92%	0.38	N/S	96%	100%	1.00	N/S	93%	100%	0.57	N/S	94%	97%	1.0000	N/S
**Presence of Geriatric Care Clinic**	92%	69%	0.052	N/S	91%	69%	0.054	N/S	**100%**	**76%**	**0.0017***	**S**	97%	76%	0.0234	N/S
**Change in Weekly COVID-19 Cases†**	4.130	3.569	0.052	N/S	6.034	5.529	0.19	N/S	6.478	5.957	0.19	N/S	0.461	0.453	0.9775	N/S

N/S indicates not significant. GoCC: Goals of care conversation

*Boldface p-value indicates statistical significance <.01 after Holm adjustment for multiple comparisons.

†For each facility, the change in the weekly COVID-19 cases on the log scale from the earlier period to the later period. The table entry is averaged over the relevant facilities.

© 2025 Linsky AM et al. *JAMA Network Open*.
