# Supplementary material for: The COVID-19 Pandemic and Goals-of-Care Conversations in Veterans Health Administration Clinics
Source: JAMA Netw Open. 2025 Jun 16;8(6):e2515980. doi: 10.1001/jamanetworkopen.2025.15980 (PMC12171934; doi:10.1001/jamanetworkopen.2025.15980)
Supplement: Supplement 2. — Data Sharing Statement [file jamanetwopen-e2515980-s002.pdf]

Data Sharing Statement

Linsky. The COVID-19 Pandemic and Goals-of-Care Conversations in Veterans Health Administration Clinics, 2019-2023. *JAMA Netw Open*. Published June 16, 2025. doi:10.1001/jamanetworkopen.2025.15980

Data
Data available: Yes
Data types: Deidentified participant data
How to access data: Requests to access de-identified data will be reviewed by the study authors and will require a signed DUA governing use of data. Requests can be made by email to the corresponding author.
When available: With publication

Supporting Documents
Document types: None

Additional Information
Who can access the data: Requests to access de-identified data will be reviewed by the study authors and will require a signed DUA governing use of data. Requests can be made by email to the corresponding author.
Types of analyses: Requests to access de-identified data will be reviewed by the study authors and will require a signed DUA governing use of data. Requests can be made by email to the corresponding author.
Mechanisms of data availability: Requests to access de-identified data will be reviewed by the study authors and will require a signed DUA governing use of data. Requests can be made by email to the corresponding author.
